# Erythrocyte Membrane Makeover by *Plasmodium falciparum* Gametocytes

**DOI:** 10.3389/fmicb.2019.02652

**Published:** 2019-11-08

**Authors:** Gaëlle Neveu, Catherine Lavazec

**Affiliations:** ^1^Inserm U1016, CNRS UMR 8104, Université de Paris, Institut Cochin, Paris, France; ^2^Laboratoire d’Excellence GR-Ex, Paris, France

**Keywords:** gametocytes, erythrocyte membrane and cytoskeleton, mechanical properties, adhesive properties, protein export

## Abstract

*Plasmodium falciparum* sexual parasites, called gametocytes, are the only parasite stages responsible for transmission from humans to *Anopheles* mosquitoes. During their maturation, *P. falciparum* gametocytes remodel the structural and mechanical properties of the membrane of their erythrocyte host. This remodeling is induced by the export of several parasite proteins and a dynamic reorganization of the erythrocyte cytoskeleton. Some of these modifications are specific for sexual stages and play a key role for gametocyte maturation, sequestration in internal organs, subsequent release in the bloodstream and ability to persist in circulation. Here we discuss the mechanisms developed by gametocytes to remodel their host cell and the functional relevance of these modifications.

## Introduction

To interact with the external environment, the parasite *Plasmodium falciparum* drastically remodels its erythrocyte host. Such modifications are mediated by the export of parasite proteins into the erythrocyte that alter the architecture of the host cell membrane. By modifying the cytoadherence, deformability and permeability properties of the host cell membrane, these proteins contribute to parasite survival, virulence, and immune evasion. All these processes are extensively described during *P. falciparum* asexual stages, however, they are less characterized in gametocytes, which are the sexual stages responsible for the transmission from humans to mosquitoes. Unlike asexual stages that replicate in a cycle of 48 h, gametocytes develop over a period of 10 days, progressing through five distinct stages of maturation (Hawking et al., 1971). Immature gametocytes from stage I to IV are absent from peripheral circulation and sequester in bone marrow parenchyma (Aguilar et al., 2014; Joice et al., 2014). The mechanisms underlying their sequestration remain poorly understood but are likely drastically different from that of asexual stages, which sequester by cytoadhesion to endothelial cells (Baruch et al., 1995; Smith et al., 1995). At maturation, erythrocytes infected with stage V gametocytes are released in the bloodstream and freely circulate for several days waiting to be taken up by mosquitoes. During this time, gametocytes should be able to circulate through the spleen and avoid immune recognition. To adapt to these different microenvironments, gametocytes express a range of proteins among which more than 10% are exported to the erythrocyte (Silvestrini et al., 2010). These proteins specifically remodel the erythrocyte membrane to allow gametocytes to interact with the host, indicating that the parasite evolved efficient strategies to renovate its host cell according to the specific needs of each life cycle phase. This review summarizes our current knowledge of the mechanisms developed by gametocytes to remodel the structural and mechanical properties of their erythrocyte host cell. We discuss the functional relevance of these modifications for gametocytes sequestration and circulation within their host.

## Protein Export at the Erythrocyte Membrane

**FIGURE 1:**
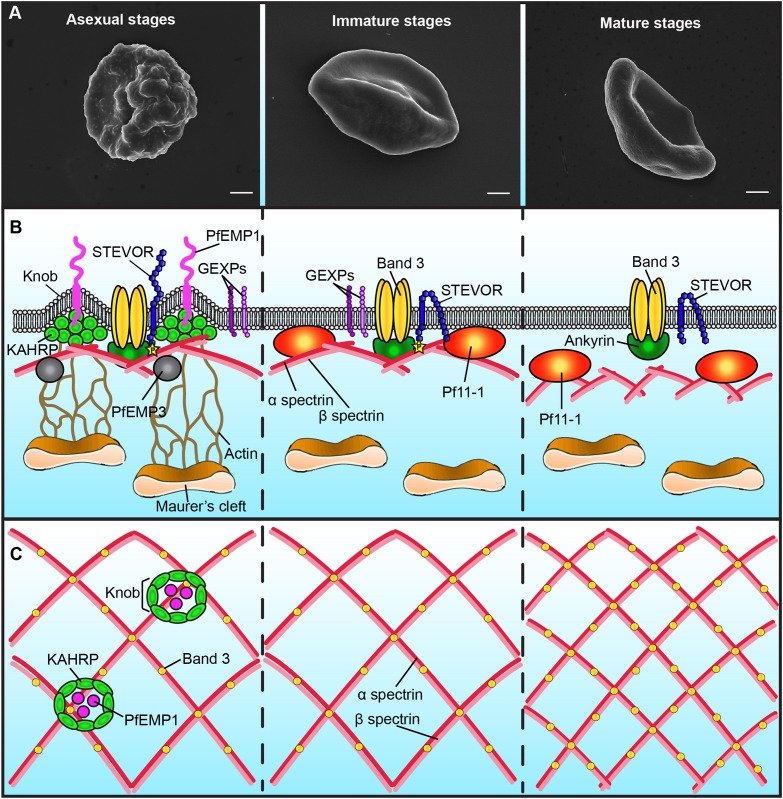
Schematic representation of the plasma membrane and cytoskeleton of *P. falciparum*-infected erythrocytes. Only major differences between different parasite stages are shown. **(A)** Scanning electron microscopy images showing the presence of knobs at the membrane of erythrocytes infected with asexual stages (left panel), but not with gametocytes (middle and right panels). Bars represent 1 μm. **(B)** Schematic representation of the infected-erythrocyte membrane. Left panel: During asexual stages the membrane of infected erythrocytes presents knobs formed by the parasite protein KAHRP. Knobs act as a scaffold for the presentation of PfEMP1 and are connected by actin filaments to the Maurer’s clefts. PfEMP3 and STEVOR interact with the spectrin network. The N-terminal domain of STEVOR is exposed at the host cell surface and the C-terminal domain is phosphorylated and linked to the ankyrin complex. GEXPs are exposed at the infected erythrocyte surface of both asexual and gametocyte stages. Middle panel: During immature gametocyte stages, knobs, PfEMP1 and KAHRP are absent from the membrane. The gametocyte-specific protein Pf11-1 is associated to the membrane. Maurer’s clefts are not tethered to the membrane by actin. The N-terminal domain of STEVOR is localized at the cytoplasmic face of the infected cell. Right panel: During the mature gametocyte stage, STEVOR is dephosphorylated and its association with the ankyrin complex is abolished. GEXPs are absent from the infected erythrocyte surface. The level of coupling between the membrane skeleton and the plasma membrane decreases. **(C)** Schematic representation of the spectrin meshwork in different parasite stages. During asexual and immature gametocyte stages the length of the spectrin cross-members and the size of the skeletal meshwork are expanded (left and middle panels). Deformable mature GIE exhibit a reduced spectrin meshwork size comparable to that in uninfected erythrocytes (right panel).

Upon infection with *P. falciparum*, the architecture of the erythrocyte membrane is deeply altered by parasite proteins (Figure 1B and Table 1). These proteins are either secreted on the erythrocyte surface by parasite apical organelles during invasion, or are exported by the developing parasite into the erythrocyte cytosol. Secreted proteins include the Ring-infected Erythrocyte Surface Antigen (RESA) contributing to the stabilization of the erythrocyte membrane skeleton (Pei et al., 2007), and the members of the RhopH protein family involved in the new permeability pathway (Counihan et al., 2017; Ito et al., 2017; Sherling et al., 2017). Many of the parasite-exported proteins possess a canonical export motif termed the *Plasmodium* export element (PEXEL) or host-targeting (HT) motif (Hiller et al., 2004; Marti et al., 2004). In addition, the *P. falciparum* exportome also includes a large number of PEXEL-negative exported proteins (PNEPs) (Heiber et al., 2013). To reach the erythrocyte cytosol, all parasite-exported proteins should pass through the parasitophorous vacuole membrane that envelops the parasite and its surrounding vacuole. Both PEXEL proteins and PNEPs cross this membrane through a protein translocon called *Plasmodium* Translocon of EXported proteins (PTEX) (de Koning-Ward et al., 2009), then transit by an exo−membranous trafficking system established by the parasite in the erythrocyte cytosol, and eventually some of them traffic further to the erythrocyte cytoskeleton and plasma membrane. Parasite proteins involved in the export machinery are expressed in both asexual and sexual stages, and proteins containing a PEXEL motif or identified as PNEPs are found at the gametocyte-infected erythrocyte membrane (Figure 1; Ingmundson et al., 2014). For instance, early studies showed that the gametocyte-specific giant protein Pf11-1 is exported to the cytoplasm of infected erythrocytes where it interacts with the erythrocyte membrane (Scherf et al., 1992). Later, comparative analysis of the proteome of asexual stages and gametocytes revealed that sexual differentiation is accompanied by an intense export of gametocyte proteins putatively involved in erythrocyte remodeling (Silvestrini et al., 2010). Some of these proteins, over-represented in early gametocytes, were called *P. falciparum* Gametocyte EXported Proteins (PfGEXP) (Silvestrini et al., 2010). The export of several PfGEXPs to the infected erythrocyte has been experimentally validated, including Pfg14.744 (Eksi et al., 2005), PfGECO (Morahan et al., 2011), PfGEXP5 (Tiburcio et al., 2015), and PfGEXP10 (Silvestrini et al., 2010). A recent report confirmed these findings and identified novel exported proteins by proteomics and immune profiling (Dantzler et al., 2019). Trypsin treatment, immunofluorescence and flow cytometry studies demonstrated erythrocyte surface exposure for six antigens, including PfGEXP7 and PfGEXP10. These proteins probably impact the properties of the gametocyte-infected erythrocyte (GIE) membrane, however, it is difficult to predict their function and their role in membrane remodeling due to the lack of any obvious functional annotation for most of the GEXPs. Several GEXPs, as PfGEXP5, belong to the PHIST (*Plasmodium* Helical Interspersed SubTelomeric) protein family (Silvestrini et al., 2010). This family of 89 exported proteins is implicated in various molecular and cellular processes (Warncke et al., 2016), and several PHIST proteins localize to the erythrocyte cytoskeleton during asexual stages where they contribute to erythrocyte remodeling (Parish et al., 2013; Oberli et al., 2016). However, since most of GEPXPs are conserved in asexual and sexual stages, they are unlikely to contribute to the specific needs of gametocytes.

**TABLE 1:** Parasite proteins involved in erythrocyte membrane makeover.

**Protein**	**Localization**	**Stage**	**Role in erythrocyte remodeling**	**References**
RESA	Erythrocyte skeleton	Asexual and sexual	Stabilization of the erythrocyte skeleton	Pei et al., 2007
RhopH	Erythrocyte membrane	Asexual	New Permeability Pathway	Counihan et al., 2017; Ito et al., 2017; Sherling et al., 2017
Pf11-1	Erythrocyte cytosol and skeleton	Sexual	Unknown	Scherf et al., 1992
Pfg14.744	Erythrocyte cytosol	Sexual	Unknown	Eksi et al., 2005
PfGECO (PfGEXP1)	Erythrocyte cytosol	Sexual	Unknown	Morahan et al., 2011
PfGEXP5	Erythrocyte cytosol	Sexual	Unknown	Tiburcio et al., 2015
PfGEXP10	Erythrocyte surface	Asexual and sexual	Antigenic exposure, adhesion	Silvestrini et al., 2010; Hermand et al., 2016; Dantzler et al., 2019
PfGEXP7	Erythrocyte surface	Asexual and sexual	Antigenic exposure, adhesion	Silvestrini et al., 2010; Hermand et al., 2016; Dantzler et al., 2019
KAHRP	Erythrocyte skeleton	Asexual	Deformability, knobs formation	Crabb et al., 1997; Glenister et al., 2002; Looker et al., 2019
PfEMP1	MC, erythrocyte surface	asexual	Adhesion, antigenic variation	Baruch et al., 1995; Chen et al., 1998
STEVOR	MC, erythrocyte membrane and surface	Asexual and sexual	Adhesion, deformability, antigenic variation	Niang et al., 2009; Sanyal et al., 2012; Tiburcio et al., 2012; Niang et al., 2014; Naissant et al., 2016; Neveu et al., 2018; Wichers et al., 2019
RIFIN	MC, erythrocyte membrane and surface	Asexual and sexual	Adhesion, immune evasion, antigenic variation	Fernandez et al., 1999; Kyes et al., 1999; Goel et al., 2015
PfEMP3	MC, erythrocyte skeleton	Asexual	Deformability	Glenister et al., 2002

*Localization, expression at asexual or sexual stages and involvement in erythrocyte remodeling are indicated with corresponding references. MC: Maurer’s Clefts.*

Major specificities of protein export in gametocytes are the absence of the Knob-Associated Histidine Protein (KAHRP) at the erythrocyte skeleton and the reduced levels of the *P. falciparum* erythrocyte membrane protein 1 (PfEMP1) exposed on the erythrocyte surface (Tiburcio et al., 2013) (Figure 1B). In contrast, the other multigenic families STEVOR and RIFIN are highly expressed during sexual differentiation (McRobert et al., 2004; Petter et al., 2008; Wang et al., 2010). Immunofluorescence studies with antibodies distinguishing A and B RIFINs revealed that only A-type RIFINs are detectable in the cytoplasm of erythrocytes infected by immature gametocytes and are localized at the erythrocyte membrane (Petter et al., 2008). STEVOR proteins have also been shown to be associated with the erythrocyte membrane in immature gametocytes stages (McRobert et al., 2004; Tiburcio et al., 2012). Although the adhesive domain of STEVOR was detected at the erythrocyte surface in asexual stages where it plays a role in rosetting (Niang et al., 2009; Niang et al., 2014; Singh et al., 2017; Wichers et al., 2019), recent immunofluorescence studies convincingly showed that this domain is not exposed at the surface of erythrocytes infected with immature gametocytes (Neveu et al., 2018). At the mature gametocyte stage, STEVOR proteins are no longer detectable at the erythrocyte membrane, probably as a result of conformational changes upon dephosphorylation (Tiburcio et al., 2012; Naissant et al., 2016; Figure 1B). Other parasite proteins also probably disappear from the membrane after stage III since a recent report highlighted that antigens exposed at the surface of stage I/II GIE, but not of mature GIE, are recognized by plasma from naturally exposed individuals (Dantzler et al., 2019). This reduced antigen expression may be due to a decreased in protein export in mature stages. Indeed, PTEX components are expressed in stage I/II gametocytes but undergo degradation during further progression of gametocyte maturation, suggesting that protein export mostly occurs in the first stages of gametocytogenesis (Matthews et al., 2013). This hypothesis would be consistent with the development of the Inner Membrane Complex from stage III onward (Dearnley et al., 2012). Indeed, this system of flattened membrane compartments underneath the gametocyte plasma membrane could represent a major obstacle for proteins to be trafficked to the erythrocyte cytosol. In addition, the reduction of protein exposure may also result from protease activity or release via extracellular vesicles. Since proteins exposed at the surface of immature GIE may be involved in the interactions with the bone marrow parenchyma, their disappearance may contribute to the release of mature GIE in the blood circulation.

## Reorganization of the Erythrocyte Plasma Membrane and Cytoskeleton

The export of hundreds of parasite proteins to the host cell and the synthesis of novel cellular structures induce a dynamic reorganization of the erythrocyte plasma membrane and of the underlying cytoskeleton. One of the major changes in the plasma membrane of erythrocytes infected with asexual stages is the appearance of thousands of small protrusions at the cell surface termed knobs that act as a scaffold for the presentation of the virulence protein PfEMP1 (Figure 1A; Luse and Miller, 1971; Langreth et al., 1978; Crabb et al., 1997). Knobs, whose formation depends on the parasite-derived protein KAHRP, consist of an electron-dense ring-shaped structure underpinned by a spiral structure and connected by multiple links to the actin-spectrin meshwork of the erythrocyte membrane skeleton (Figure 1C; Crabb et al., 1997; Looker et al., 2019). Scanning and transmission electron microscopy analyses revealed that the surface of erythrocytes infected by developing gametocytes from stage II to V are devoid of knobs (Figure 1A; Sinden, 1982; Tiburcio et al., 2012). In contrast, an early study reported that knobs are present at the surface of stage I GIE (Day et al., 1998). Of note, this study did not benefit from state-of-the-art tools to distinguish asexual from early sexual stages and rather used standard light microscopy for parasite stages differentiation. More recently, transgenic parasites expressing a fluorescent reporter under a gametocyte-specific promoter allowed separation of stage I gametocytes from asexual trophozoites (Silvestrini et al., 2010). Electron microscopy analysis of purified early gametocytes from this transgenic line established that stage I gametocytes do not modify their surface with knob structures (Tiburcio et al., 2013). The erythrocyte membrane modifications induced by gametocyte development rather occur in the cytoskeleton, with remodeling of both lateral and vertical interactions within the skeleton (Dearnley et al., 2016). As observed in asexual stages (Shi et al., 2013), atomic force microscopy of the cytoplasmic surface of the membrane of mechanically sheared infected erythrocytes revealed that the length of the spectrin cross-members and the size of the skeletal meshwork increase in developing immature GIE (Figure 1C; Dearnley et al., 2016). This expansion is accompanied by enhanced coupling of the membrane skeleton to the membrane bilayer in immature stages, as evidenced by an altered lateral mobility of Band 3 monitored by microscope-based photobleaching (Parker et al., 2004; Dearnley et al., 2016). In parallel, erythrocytic actin is relocated from the skeleton to the parasite-derived organelles known as Maurer’s Clefts (Dearnley et al., 2016). However, in the absence of knobs, which act as anchoring points for these organelles in asexual stages, actin relocation does not tether Maurer’s Clefts onto the erythrocyte membrane in gametocytes, thereby increasing their mobility (Cyrklaff et al., 2011; Dearnley et al., 2016). All these modifications are then reversed in stage V GIE, which exhibit a spectrin length and a Band 3 mobile fraction similar to uninfected erythrocytes (Figure 1C). A composite model predicting the physical consequences of restructuring the skeletal meshwork revealed that these reversible changes are likely linked to the switch in GIE deformability occurring at the transition from immature to mature gametocyte stages (Tiburcio et al., 2012; Dearnley et al., 2016).

## Changes in Erythrocyte Deformability

The profound reorganization of the membrane nanostructure deeply affects the mechanical properties of the infected erythrocyte membrane. During *P. falciparum* asexual development, the infected erythrocyte becomes progressively rigid and the erythrocyte membrane loses its elasticity (Cranston et al., 1984; Nash et al., 1989), in part due to the export of parasite proteins that interact with the erythrocyte skeleton, including KAHRP, PfEMP3, RESA and STEVOR (Figure 1B and Table 1; Glenister et al., 2002; Mills et al., 2007; Sanyal et al., 2012). Similar biophysical changes occur during gametocytogenesis: an increase in rigidity is observed upon infection with immature gametocytes from stage I to IV, followed by a switch in deformability at the transition between stage IV and stage V (Aingaran et al., 2012; Dearnley et al., 2012; Tiburcio et al., 2012). Increased stiffness of immature GIE may contribute to their sequestration in the bone marrow parenchyma by mechanical retention, whereas the newly acquired deformability of mature GIE leads to a restored ability to cross narrow apertures and may help them survive in the circulation, where they can be picked up by mosquitoes. The regulation of GIE deformability results from a combination of several factors that are beginning to be elucidated. While it has long been thought that immature GIE stiffness was fully dependent on the gametocyte microtubule skeleton, a study reported that disruption of the microtubule network did not influence immature GIE rigidity (Dearnley et al., 2016). These observations suggest that regulation of GIE deformability mainly results from the parasite-induced modifications of its host erythrocyte rather than from the parasite cytoskeleton. In contrast, treatment of immature GIE with cytochalasin D increased GIE deformability, suggesting an important role of the actin cytoskeleton in the GIE membrane stiffness (Dearnley et al., 2016).

As shown in asexual stages, GIE membrane viscoelasticity is affected by the export of parasite proteins to the erythrocyte membrane skeleton. For instance, modulation of GIE deformability is dependent on presence of the parasite proteins STEVOR at the erythrocyte membrane (Tiburcio et al., 2012) and on the interaction of the cytoplasmic domain of STEVOR with erythrocyte proteins composing the ankyrin complex (spectrin α, spectrin β, ankyrin and Band 3) (Figure 1B; Naissant et al., 2016). This interaction is dependent on PKA-mediated phosphorylation of the STEVOR cytoplasmic tail and is tightly regulated by the parasite. STEVOR phosphorylation only occurs in immature stages and is due to increased cAMP levels resulting from a low expression of the plasmodial phosphodiesterase δ (PfPDEδ) at this stage. Rising expression of PfPDEδ in mature stages leads to a drop of cAMP levels that reduce the phosphorylation of both STEVORs and their cytoskeletal partner(s) and consequently weaken or abolish their interactions, eventually resulting in an increase of infected erythrocyte deformability (Ramdani et al., 2015; Naissant et al., 2016). In accordance with the key role of PfPDEδ in this mechanism, the marketed PDE inhibitor sildenafil (Viagra^®^) increases cAMP concentration in mature GIE and impairs their circulation in an *in vitro* model for splenic retention (Ramdani et al., 2015). In line with these observations, treatment of *P. berghei*-infected mice with sildenafil increases gametocytes homing to sequestration sites, suggesting that PDE inhibitors may cause gametocytes to become mechanically trapped in the bone marrow and splenic cords (De Niz et al., 2018).

## Remodeling of Adhesives Properties

To sequester away from the peripheral circulation, asexual parasites deeply modify the adhesive properties of their host erythrocyte membrane and adhere to the host microvasculature via specific ligand-receptor interactions, primarily mediated by the parasite antigen PfEMP1 (Baruch et al., 1995). This adhesin is anchored on knobs at the surface of infected erythrocytes and binds to host endothelial receptors such as ICAM-1 and CD36 (Figures 1B,C; Miller et al., 2002). Erythrocytes infected with asexual parasites could also adhere to surrounding non-infected erythrocytes through binding of different antigenic variants of the parasite (PfEMP1, STEVOR and RIFIN) to different receptors on the surface of the erythrocyte, leading to rosette formation (Chen et al., 1998; Niang et al., 2014; Goel et al., 2015). Unlike asexual stages, the adhesive properties of the GIE membrane have long been debated and are still elusive. Early studies produced conflicting data on GIE adhesion efficiency and reported low binding of immature GIE, but not of mature stages, to C32 melanoma or bone marrow stromal cell lines (Rogers et al., 1996; Rogers et al., 2000). This conclusion was, however, not confirmed by another report showing that stage I GIE adhere to CD36-expressing C32 melanoma cells while stage II to V GIE lose their adhesive properties (Day et al., 1998). More recent studies have convincingly shown that immature stage I-IV GIE do not adhere to a panel of endothelial cell lines from various human organs (Silvestrini et al., 2012; Tiburcio et al., 2013). These results are in accordance with the detection of immature gametocytes in extravascular spaces of bone marrow in *ex vivo* and autopsy specimens from malaria-infected patients (Farfour et al., 2012; Joice et al., 2014). The unveiling of the hidden sites for gametocytes maturation led to the hypothesis that they could adhere to non-endothelial bone marrow cells. Recent work using an *in vitro* tridimensional co-culture system revealed that immature GIE adhere to human bone marrow mesenchymal stem cells via trypsin-sensitive parasite ligands exposed on the erythrocyte surface (Messina et al., 2018). This adhesion induces stimulation of mesenchymal cells to secrete a panel of cytokines and growth factors involved in angiogenesis, thus suggesting a mechanism used by the parasite to remodel the bone marrow endothelium. It has been proposed that the GIE surface proteins GEXP7 and GEXP10, which are able to bind CX3CL1, a chemokine expressed on bone marrow stromal cells, may be involved in this adhesion (Hermand et al., 2016; Messina et al., 2018). However, their insensitivity to trypsin treatment does not support this hypothesis (Dantzler et al., 2019). Although the ligand(s) and receptor(s) promoting such interactions are not identified yet, this mechanism may contribute to immature GIE sequestration in the parenchyma. In contrast, cell-cell adhesion assays with human primary erythroblasts revealed that immature GIE do not specifically adhere to erythroid precursors, despite the fact that some parasite antigens involved in rosetting of asexual stages are expressed by gametocytes and may bind to their receptors present at the surface of erythroblasts (Neveu et al., 2018). The absence of a rosetting-like phenotype in sexual stages is likely due to the reduced levels of PfEMP1 (Tiburcio et al., 2013) and to the failure to detect STEVOR at the surface of immature GIE (Neveu et al., 2018). These observations highlighted that some parasite adhesins may have different role and topology in asexual and sexual stages, which may reflect a strategy that the parasite has evolved to differently modify its host cell to adapt to different microenvironments (Neveu et al., 2018).

## Concluding Remarks

Recent studies have greatly improved our understanding of how *P. falciparum* gametocytes redecorate the surface of their erythrocyte host with parasite antigens and how they remodel the erythrocyte deformability and adhesive properties. Many questions remain outstanding regarding the sequestration mechanisms of immature gametocytes in the bone marrow parenchyma and how erythrocyte remodeling contributes to these processes. The increase in erythrocyte membrane stiffness and the export of adhesive molecules at the erythrocyte surface may contribute to immature GIE sequestration by mechanical retention and by adhesion to bone marrow cells, respectively. Besides these mechanisms, the maintenance of immature gametocytes in the bone marrow parenchyma may also be mediated by the infection of erythroblasts constituting the erythroblastic islands. In support of this hypothesis, the development of asexual parasites can take place in a culture of human erythroblasts and immature gametocytes of stages I and II have been observed in these cells (Tamez et al., 2009; Joice et al., 2014). Whether gametocytes can complete their development in erythroblasts and how the parasite remodels these nucleated cells remains to be investigated.

Our current understanding of the host cell remodeling induced by *P. falciparum* gametocytes opens avenues toward the design of novel interventions to interrupt parasite transmission and the spread of malaria, including transmission-blocking drugs targeting GIE mechanical properties and transmission-blocking vaccines targeting GIE surface antigens.

## Author Contributions

GN drafted the manuscript. CL edited the manuscript.

## Conflict of Interest

The authors declare that the research was conducted in the absence of any commercial or financial relationships that could be construed as a potential conflict of interest.
